# Association of KCNQ1rs2237892C⟶T Gene with Type 2 Diabetes Mellitus: A Meta-Analysis

**DOI:** 10.1155/2021/6606830

**Published:** 2021-11-22

**Authors:** Wen-Jia Han, Jian-Yi Deng, Hua Jin, Li-Ping Yin, Jin-Xia Yang, Jiang-Jie Sun

**Affiliations:** ^1^School of Dentistry, Anhui Medical University, Hefei, Anhui 230032, China; ^2^Clinical Medical College, Anhui Medical University, Hefei, Anhui 230032, China; ^3^Inner Mongolia University For Nationalities, Affiliated Hospital, Nationalities, 028000, China; ^4^Medical Examination Center, The First Affiliated Hospital of Anhui Medical University, Hefei 230022, China; ^5^Health Management College, Anhui Medical University, Hefei, Anhui 230032, China

## Abstract

**Background:**

Type 2 diabetes mellitus (T2DM) is one of the most common chronic diseases in adults, causing high morbidity and mortality worldwide. In recent years, the prevalence of T2DM has been increasing significantly, and genome-wide association studies (GWAS) have shown that KCNQ1 significantly increases the risk of T2DM.

**Objective:**

To find large-scale evidence on whether the KCNQ1rs2237892C⟶T gene polymorphism is associated with T2DM susceptibility.

**Methods:**

A comprehensive review of the Chinese and English literature on the association of T2DM with KCNQ1rs2237892 is published by PubMed and Baidu Academic. The included literature was part or all of the studied loci which were evaluated for association with T2DM. Forest plots were made of the included literature to analyze the association of KCNQ1 with polymorphisms of the studied loci, and funnel plots and Egger's test were used to evaluate the publication bias of the selected included literature.

**Results:**

Ten case-control studies including a total of 7027 cases and 8208 controls met our inclusion criteria. Allele (C allele frequency distribution) (OR: 1.19; 95% CI: 0.87,1.62; *P* < 0.00001), recessive (OR: 0.73; 95% CI: 0.45,1.18; *P* < 0.00001) genetic model under the full population was observed between KCNQ1rs2237892C⟶T gene polymorphism and T2DM without a significant relationship. In a stratified analysis by race, a meaningful association was found in non-Asian populations under the allelic genetic model, but no association was found in Asian populations.

**Conclusion:**

This meta-analysis showed no significant association between the rs2237892 polymorphism of the KCNQ1 gene and the risk of T2DM.

## 1. Introduction

Type 2 diabetes mellitus (T2DM) is a genetically heterogeneous metabolic disorder characterized by chronic hyperglycemia due to impaired insulin secretion and sensitivity influenced by genetic and environmental factors. Patients with T2DM are often associated with macrovascular disease, diabetic retinopathy, diabetic nephropathy, and diabetic neuropathy [1]. The Global Diabetes Map (9th edition) published by IDF-2019 shows that an estimated 463 million people aged 20-79 years currently have diabetes worldwide, the vast majority with type 2 diabetes, with 578 million and 700 million expected in 2030 and 2045 [2]. People around the world suffer from type 2 diabetes and its complications. Currently, the etiology of T2DM is unknown, and two independent genome-wide association studies suggest that KCNQ1 is a novel gene susceptible to T2DM [3, 4]. KCNQ1 is commonly expressed in epithelial cells, and KCNQ1 expression was also observed in insulin-secreting INS-1 cells. The selective inhibitor chromanol 293B inhibited KCNQ1 potassium channel activity and significantly increased insulin secretion [5]; Yazdi et al. demonstrated a significant association between KCNQ1 and T2DM [6], but, research showed there was no significant association between KCNQ1 and T2DM [7]; Liu et al. and Yu et al. performed meta-analysis of multiple loci in the KCNQ1 gene, and the results again demonstrated the association between KCNQ1 and T2DM [8, 9], but these two papers introduced data from meta-analyses, respectively, and their strength of proof may be weakened.

Therefore, we performed this meta-analysis to further demonstrate whether genetic factors play a crucial role in the pathogenesis of T2DM.

## 2. Materials and Methods

### 2.1. Literature Search

The advanced search of the literature search library was conducted by using “T2DM, KCNQ1, rs2237892” as the search term in China National Knowledge Infrastructure (CNKI) and Baidu Academic with the following search formula: subject (T2DM) and keyword (KCNQ1) and keyword (rs2237892). English literature was obtained for case-control studies and cohort studies on the association of KCNQ1 gene polymorphism with T2DM. The last search was conducted on June 12, 2021. Inclusion criteria were (i) meeting the diagnostic criteria for diabetes mellitus published by the World Health Organization in 1990 or ADA in 2010; (ii) the study type was a case-control study; (iii) there were year of publication; there were clear regulations on sample content; the study results were informative enough for analyzing whether the differences in genotypes and alleles between the case and control groups were statistically significant; (iv) the control groups all met the H-W genetic equilibrium pattern; (v) sources of information on control and case groups were provided; and (vi) patients were randomly selected, with no special restrictions on age, sex, or family history. The exclusion criteria were (i) lack of sufficient control group; (ii) exclusion of literature review; (iii) exclusion of studies with gestational diabetes as an endpoint; and (iv) insufficient sample size.

### 2.2. Data Extraction

**Figure 1:**
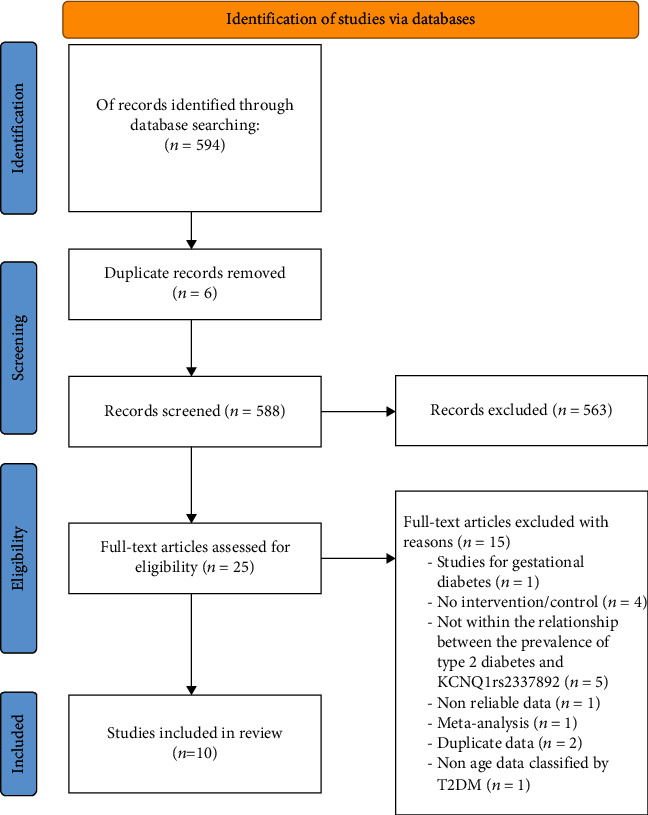
Literature screening process.

Two investigators independently performed literature reading and information extraction from the eligible literature based on exclusion and inclusion criteria. When ambiguities were encountered, agreement was eventually reached in whether to extract data from the papers by conferring with the third investigator. For each paper, the following paper information was collected: (i) author's name; (ii) year of publication; (iii) country; (iv) number of included cases and controls; (v) allele and genotype data; and (vi) mean age of included cases and controls. The literature screening process is shown in Figure 1.

### 2.3. Statistical Analysis

Statistical analysis was completed using RevMan 5 software, and the OR values and their corresponding 95% CIs were used as criteria for data statistics to compare rs2237892 allele distributions. Allelic (KCNQ1rs2237892C⟶C allele frequency distribution of T gene polymorphism), recessive (TT vs. CC+CT) genetic models were used, and the significance level was set at *P* < 0.05. If there was heterogeneity between individual studies (*I*^2^ > 50%), a random effects model was used to calculate combined effect estimates; otherwise, a fixed effects model was used, and the *Z* test was used to determine combined OR significance. Potential publication bias was estimated using funnel plots. The degree of asymmetry was statistically assessed using the Egger unweighted regression asymmetry test using STATA 11.0 software.

## 3. Results

### 3.1. Literature Search

We obtained studies on association between diabetes and gene locus polymorphism from Baidu Academic and China Knowledge Network, and some literature had duplicate publications, such as, double submission in Chinese and English, multiple submissions in one manuscript, and overlapping databases. After reading the titles and abstracts of the papers for the first screening, and reading the full text for the second screening, 10 papers were finally included. The required data were recorded by reading the full text to form a dataset for meta-analysis. The number of T2DM patients in the group included in the meta-analysis was 7027, and the number of controls was 8208, with 8 datasets for studies originating from Asia and 2 datasets for studies originating from non-Asia. Information on the first author and year of publication, sample size, mean age of control and case groups, genotype data, and matching criteria for each study are shown in Table 1.

### 3.2. Meta-Analysis

**Figure 2:**
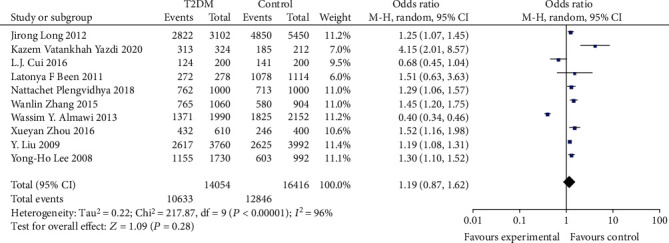
Forest plot of meta-analysis of the association between KCNQ12237892 locus and T2DM under the allele model.

**Figure 3:**
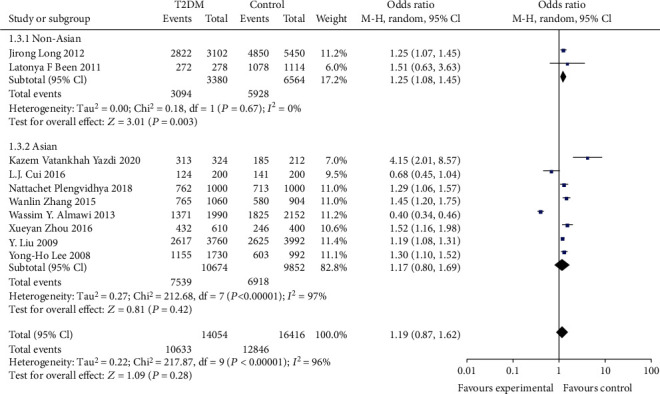
Forest plot of meta-analysis of the association between KCNQ12237892 locus and T2DM under the allele model (stratified analysis).

**Figure 4:**
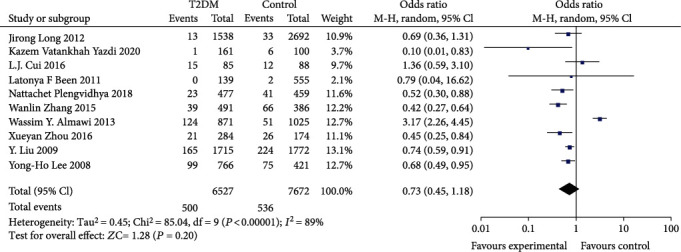
Forest plot of meta-analysis of the association between KCNQ12237892 locus and T2DM under the recessive model.

**Figure 5:**
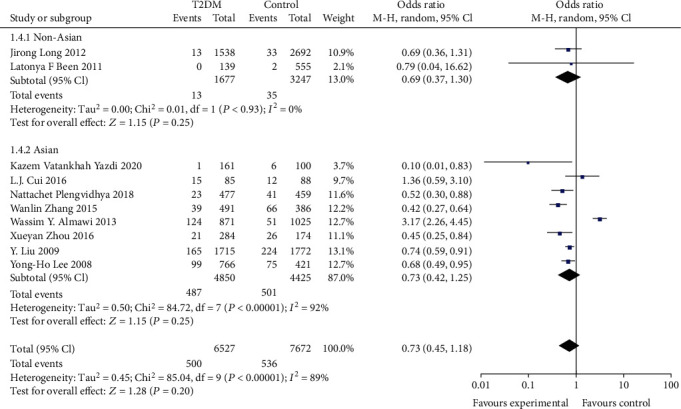
Forest plot of meta-analysis of the association between KCNQ12237892 locus and T2DM under the recessive model (stratified analysis).

In assessing the effect of the KCNQ1 rs2237892 locus polymorphism on the occurrence of T2DM susceptibility, a total of 10 studies were included in the meta-analysis after a literature data search. The association between T2DM risk and rs2237892 locus polymorphism was assessed using recessive model and allelic model, respectively. Most of the populations were from Asia, so stratified analysis was performed for Asian and non-Asian populations, and the results are shown in Figures 2–5.

We found significant heterogeneity in the total population and Asian subgroups, and non-Asian populations reflected significant homogeneity (see Table 2), so a random effects model was chosen. No significant relationship between allele (OR: 1.19; 95% CI:0.87, 1.62; *P* = 0.28) and recessive (OR: 0.73; 95% CI: 0.45, 1.18; *P* = 0.25) genetic model was observed between KCNQ1rs2237892C⟶T gene polymorphism and T2DM in the total population. In subgroup analysis stratified by race, individuals carrying the C allele in the Asian subgroup were not associated with T2DM incidence observed under the allelic (OR: 1.17; 95% CI: 0.80, 1.69; *P* = 0.42) genetic model, and recessive (OR: 0.73; 95% CI: 0.42-1.25; *P* = 0.25) genetic model individuals with CT+TT genotype in the Asian subgroup were not significantly predisposed to increased risk of T2DM. The recessive (OR: 0.69; 95% CI: 0.37, 1.30; *P* = 0.25) genetic model observed no association between CT+TT genotype and T2DM incidence in non-Asian subgroups. However, a significantly increased risk of T2DM patients carrying the C allele was observed in the non-Asian subgroup under the allelic (OR: 1.25; 95% CI: 1.08, 1.45; *P* = 0.003) genetic model.

## 4. Discussion

In Asian populations, two independent GWASs have identified KCNQ1 as a susceptibility gene for T2DM, but various studies have shown conflicting results.

The KCNQ1 gene, a member of the voltage-dependent potassium channel family, is located on chromosome 11 at 11p15.5 and expressed primarily in the heart and less frequently in the pancreas, placenta, lung, liver, kidney, brain, and adipose tissue. Mutations in KCNQ1 cause K+ channel dysfunction, which leads to cardiac long QT syndrome, familial atrial fibrillation, etc. In addition, its mutation has been found to be associated with hearing loss. T2DM development and progression are characterized by insulin resistance and islet *β*-cell dysfunction as the main pathophysiological features, and islet *β*-cell dysfunction is the determinant of DM development. Mutations in any specific protein gene involved in glucose recognition, insulin processing, or secretion can lead to islet *β*-cell dysfunction. Based on previous studies, it is hypothesized that regulation of potassium channels increases KCNQ1 protein expression on pancreatic *β*-cells, which reduces insulin secretion and raises blood glucose levels. However, the contribution of KCNQ1 to the pathogenesis of type 2 diabetes is currently unclear [10].

In this paper, we performed allelic model and recessive gene model meta-analysis of the 10 included studies on the KCNQ1rs2237892 locus and T2DM. Meta-analysis revealed no significant differences in the frequencies of the three genotypes (CC, CT, and TT) and two alleles (C and T) of KCNQ1 rs2237892SNP in the case and control groups. The finding is inconsistent with previous studies in which Japanese researcher first found a statistically significant association between the KCNQ1 rs2237892 locus and T2DM onset in an Asian population in a whole gene chain scan study [4] and did not conclude a correlation between KCNQ1 rs2237892 locus and T2DM susceptibility in our study. It has been demonstrated in several independent studies that for Asian populations, variants in the KCNQ1 rs2237892 locus confer susceptibility to T2DM. rs2237892, rs2237895, and rs2237897 were in a block of linkage disequilibrium, as confirmed by Liu et al. using a conditional independent effects test. rs2237892 and rs2237895 on type 2 diabetes can be attributed to rs2237897 [11]. A study confirmed that the C allele increased the risk of type 2 diabetes in a Chinese Han population, replicating the association of KCNQ1 rs2237892 with T2DM susceptibility, and their study also indicated that genotype CC tended to be associated with an increased risk of hypertension and macrovascular complications in patients with T2DM [1], and Zhou et al. presented new data suggesting that KCNQ1 polymorphisms affect T2DM risk by regulating the IRS-2/PI(3)K/Akt signaling pathway [12]. Nattachet et al. found a significant correlation between rs2237892SNP and T2D under additive and recessive models [13]. It was proved that rs2237892SNP is associated with T2DM in Korean population and in Lebanese population [14, 15], but Cui et al. suggested that Chinese Kazakhs rs2237892SNP may not be associated with T2DM [7], which is consistent with our findings. The frequency of KCNQ1 rs2237892SNP in Asians noted by Liu et al. is lower than that in Europeans (92-96%), and the gene KCNQ1 rs2237892SNP was not significantly associated with T2D in the original European genome-wide association study. While the high percentage of non-Asian population and low percentage of Asians in our study may be one of the reasons for the lack of significant association between KCNQ1 rs2237892SNP and T2DM, the second possible reason is that Yu et al. conducted meta-analysis on multiple sites of KCNQ1 gene and further collected and processed on the basis of meta-analysis data; there are cases of attenuated output. The third possible reason lies in the higher conditions of data collection for this meta-analysis, which improved the data quality but also had the limitation of insufficient sample size. Of course, other possibilities are not excluded.

In conclusion, our investigation cannot prove the association of KCNQ1 rs2237892SNP with T2DM in Asian populations, and the observation of the C allele as a risk allele for T2DM in non-Asian populations may be due to small sample sizes [16, 17], taking into account that geographical or cultural barriers increase the genetic distance, functional genes or regulatory regions may be disturbed by independent sets of rare mutations, and different patterns of linkage disequilibrium may also be an influencing factor.

In our study, we did not find that the KCNQ1rs2237892 gene polymorphism was associated with T2DM risk, which is inconsistent with the results of other studies. This apparent discrepancy may be mainly due to the different genotype and allele frequencies of KCNQ1rs2237892 in populations with different clinical characteristics, geographic distribution, and ethnic origin. However, the current meta-analysis has some limitations. Large-scale studies on the association of T2DM with the KCNQ1rs2237892 locus are still lacking, and therefore, a larger sample size is needed for further studies in which consideration of all races of subjects may be beneficial to validate the exact results of such observations. These findings may fill a gap in the knowledge system of genetic variation and T2DM-related studies, and therefore, genome-wide association studies in multiple races and geographic regions are necessary.

A possible limitation of this study is that the sample size is small, and there may be bias in the results. Our later study will likely increase the sample size and further explore this issue using a stratified cross-sectional design.

## 5. Conclusion

We concluded that there was no significant association between the rs2237892 polymorphism of the KCNQ1 gene and the risk of T2DM in a meta-analysis of 10 case-control studies from Asia and non-Asia including a total of 7027 cases and 8208 controls.

## Figures and Tables

**Table 1:** Characteristics of KCNQ1 gene rs2237892 polymorphism and T2DM gene association study.

Author (year)	Ethnicity	No. of case/control	Age of case/control	Matching criteria	Control			T2DM		
					CC	CT	TT	CC	CT	TT
Y. Liu (2009)	China	1,880/1,996	63.9 ± 9.5/58.1 ± 9.4	Ethnicity	853	919	224	902	813	165
Latonya F Been (2011)	US-India	139/557	48.0 ± 13.5/48.0 ± 13.5	Ethnicity	523	32	2	133	6	0
Wanlin Zhang (2015)	China	530/452	60.95 ± 12.62/58.83 ± 11.40	Ethnicity	194	192	66	274	217	39
Xueyan Zhou (2016)	China	305/200	48.93 ± 11.89/50.07 ± 6.42	Ethnicity	72	102	26	148	136	21
L. J. Cui (2016)	China	100/100	51.21 ± 11.60/49.85 ± 12.41	Ethnicity	53	35	12	39	46	15
Nattachet Plengvidhya (2018)	Thailand	500/500	53.0 ± 8.4/57.2 ± 12.2	Ethnicity, sex	254	205	41	285	192	23
Kazem Vatankhah Yazdi (2020)	Iran	162/106	65 ± 7.5/65.5 ± 7.3	Ethnicity	85	15	6	152	9	1
Yong-Ho Lee (2008)	Korea	865/496	58.2 ± 11.1/55.0 ± 9.4	Ethnicity	182	239	75	389	377	99
Jirong Long (2012)	African American	1,551/2,725	59.7 ± 8.7/57.7 ± 9.0	Ethnicity, sex	2158	534	33	1284	254	13
Wassim Y. Almawi (2013)	Lebanon	995/1076	58.6 ± 13.4/57.3 ± 10.4	Ethnicity	800	225	51	500	371	124

**Table 2:** Meta-analysis in association between KCNQ1rs2237892C→T gene in different population.

KCNQ1	Group	A fixed-effects model			A random-effects model			Heterogeneity		
		OR (95%CL)	*Z*	*P*	OR (95%CL)	*Z*	*P*	*χ* ^2^	*I* ^2^ (%)	*PQ*-test
Distribute of C allelic frequency	Total	1.07 [1.01, 1.13]	2.24	0.03	1.19 [0.87, 1.62]	1.09	0.28	217.87	96	<0.00001
	Asian	1.04 [0.98, 1.10]	1.15	0.25	1.17 [0.80, 1.69]	0.81	0.42	212.68	97	<0.00001
	Non-Asian	1.25 [1.08, 1.45]	3.02	0.003	1.25 [1.08, 1.45]	3.01	0.003	0.18	0	0.67

TT vs. CC+TC	Total	0.85 [0.74, 0.96]	2.56	0.01	0.73 [0.45, 1.18]	1.28	0.20	85.04	89	<0.00001
	Asian	0.85 [0.75, 0.97]	2.36	0.02	0.73 [0.42, 1.25]	1.15	0.25	84.72	92	<0.00001
	Non-Asian	0.69 [0.37, 1.30]	1.15	0.25	0.69 [0.37, 1.30]	1.15	0.25	0.01	0	0.93

## Data Availability

The data used to support the findings of this study are included within the article.

## Supplementary Materials

Supplementary Materials Supplementary Figure 6: funnel plot of meta-analysis of the association between KCNQ12237892 locus and T2DM under the allele model. Supplementary Figure 7: funnel plot of meta-analysis of the association between KCNQ12237892 locus and T2DM under the allele model (stratified analysis). Supplementary Figure 8: funnel plot of meta-analysis of the association between KCNQ12237892 locus and T2DM under the recessive model. Supplementary Figure 9: funnel plot of meta-analysis of the association between KCNQ12237892 locus and T2DM under the recessive model (stratified analysis).
