# Supplementary material for: Association of KCNQ1rs2237892C⟶T Gene with Type 2 Diabetes Mellitus: A Meta-Analysis
Source: J Diabetes Res. 2021 Nov 22;2021:6606830. doi: 10.1155/2021/6606830 (PMC8629679; doi:10.1155/2021/6606830)
Supplement: Supplementary Materials — Supplementary Figure 6: funnel plot of meta-analysis of the association between KCNQ12237892 locus and T2DM under the allele model. Supplementary Figure 7: funnel plot of meta-analysis of the association between KCNQ12237892 locus and T2DM under the allele model (stratified analysis). Supplementary Figure 8: funnel plot of meta-analysis of the association between KCNQ12237892 locus and T2DM under the recessive model. Supplementary Figure 9: funnel plot of meta-analysis of the association between KCNQ12237892 locus and T2DM under the recessive model (stratified analysis). [file 6606830.f1.zip › Final Publication bias.docx]

**Publication bias**

There was no significant publication bias in the current meta-analysis. To perform egger' test for publication bias, p=0.726 under the allelic model and p=0.603 under the recessive genetic model, both with p values much greater than 0.05, suggesting no publication bias. Funnel plot analysis is based on whether the graph is symmetrical to determine whether there is bias in the Meta-analysis. There are often multiple reasons for the asymmetry of the funnel plot such as: lack of rigor in the process from study design to study conclusion, small study sample size, and the presence of random error, etc. Observing Figure 6-9, it can be found that there is no significant publication bias in the funnel plot.

Fig 6

Fig 6 Funnel plot of meta-analysis of the association between KCNQ12237892 locus and T2DM under the allele model

Fig 7

Fig 7 Funnel plot of meta-analysis of the association between KCNQ12237892 locus and T2DM under the allele model(Stratified analysis)

Fig 8

Fig 8 Funnel plot of meta-analysis of the association between KCNQ12237892 locus and T2DM under the recessive model

Fig 9

Fig 9 Funnel plot of meta-analysis of the association between KCNQ12237892 locus and T2DM under the recessive model(Stratified analysis)
