# Supplementary figures and images for: Association of KCNQ1rs2237892C⟶T Gene with Type 2 Diabetes Mellitus: A Meta-Analysis
Source: J Diabetes Res. 2021 Nov 22;2021:6606830. doi: 10.1155/2021/6606830 (PMC8629679; doi:10.1155/2021/6606830)

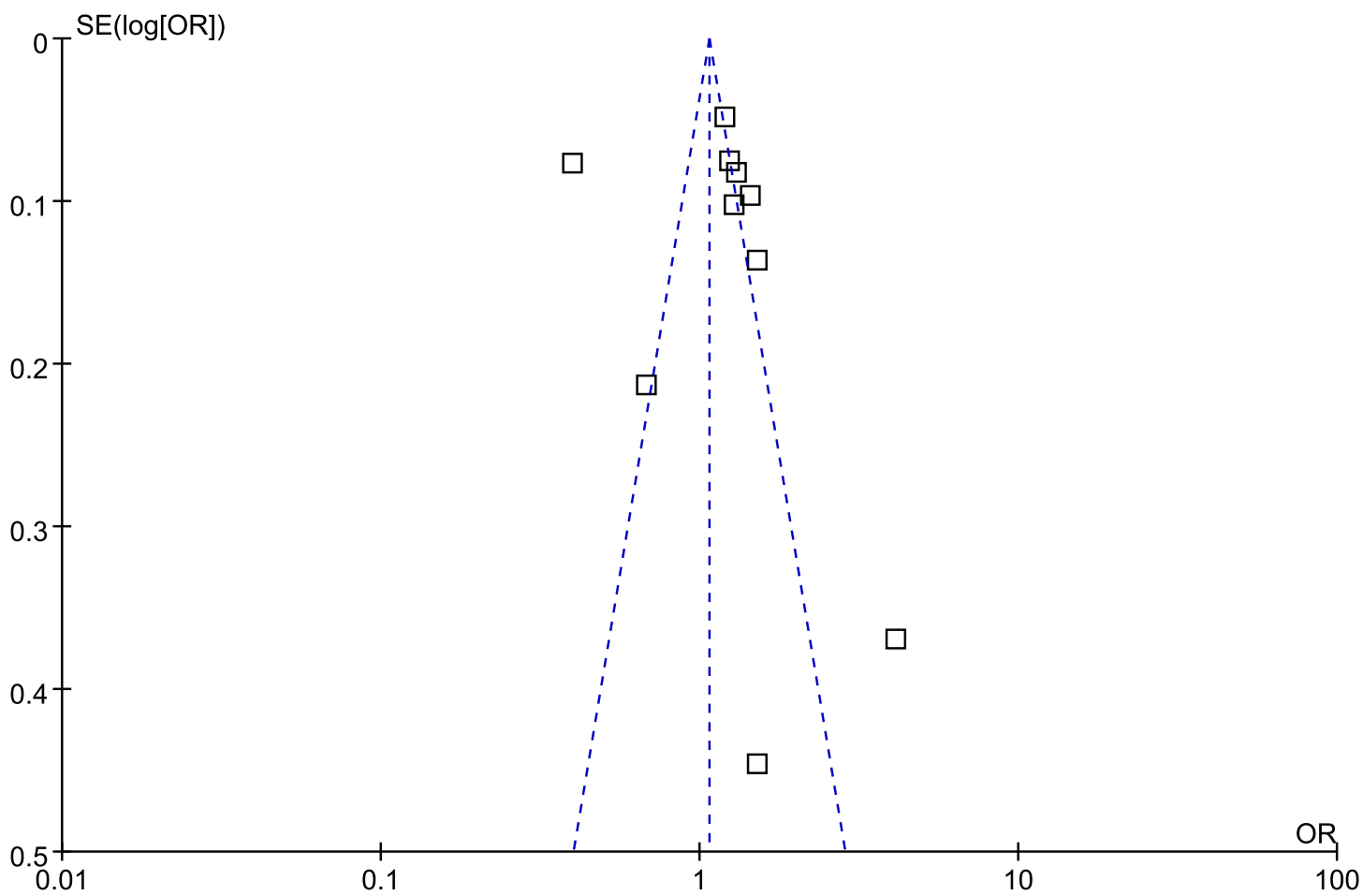

Supplement: Supplementary Materials — Supplementary Figure 6: funnel plot of meta-analysis of the association between KCNQ12237892 locus and T2DM under the allele model. Supplementary Figure 7: funnel plot of meta-analysis of the association between KCNQ12237892 locus and T2DM under the allele model (stratified analysis). Supplementary Figure 8: funnel plot of meta-analysis of the association between KCNQ12237892 locus and T2DM under the recessive model. Supplementary Figure 9: funnel plot of meta-analysis of the association between KCNQ12237892 locus and T2DM under the recessive model (stratified analysis). [file 6606830.f1.zip › Fig6 (1).pdf]

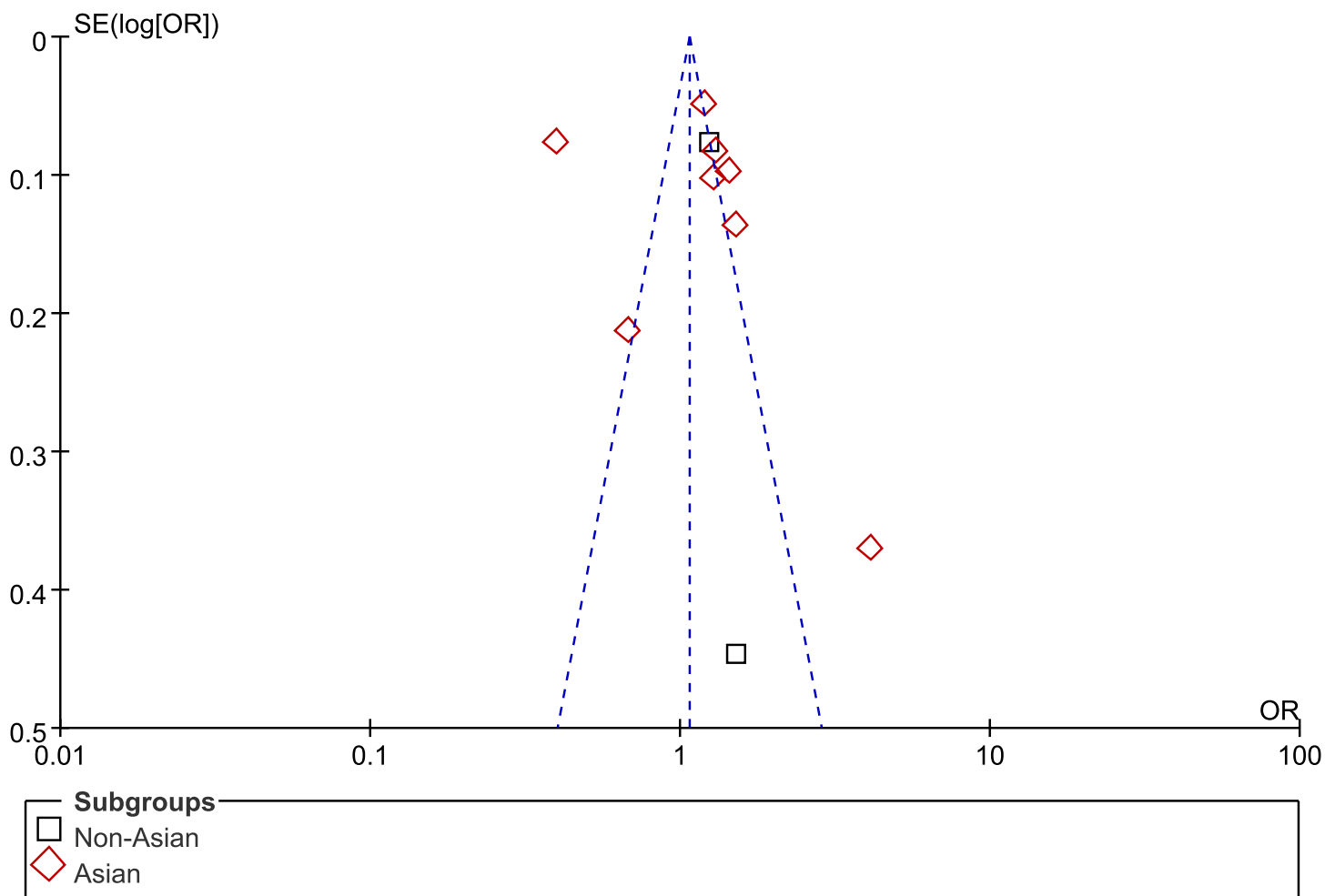

Supplement: Supplementary Materials — Supplementary Figure 6: funnel plot of meta-analysis of the association between KCNQ12237892 locus and T2DM under the allele model. Supplementary Figure 7: funnel plot of meta-analysis of the association between KCNQ12237892 locus and T2DM under the allele model (stratified analysis). Supplementary Figure 8: funnel plot of meta-analysis of the association between KCNQ12237892 locus and T2DM under the recessive model. Supplementary Figure 9: funnel plot of meta-analysis of the association between KCNQ12237892 locus and T2DM under the recessive model (stratified analysis). [file 6606830.f1.zip › Fig7 (1).pdf]

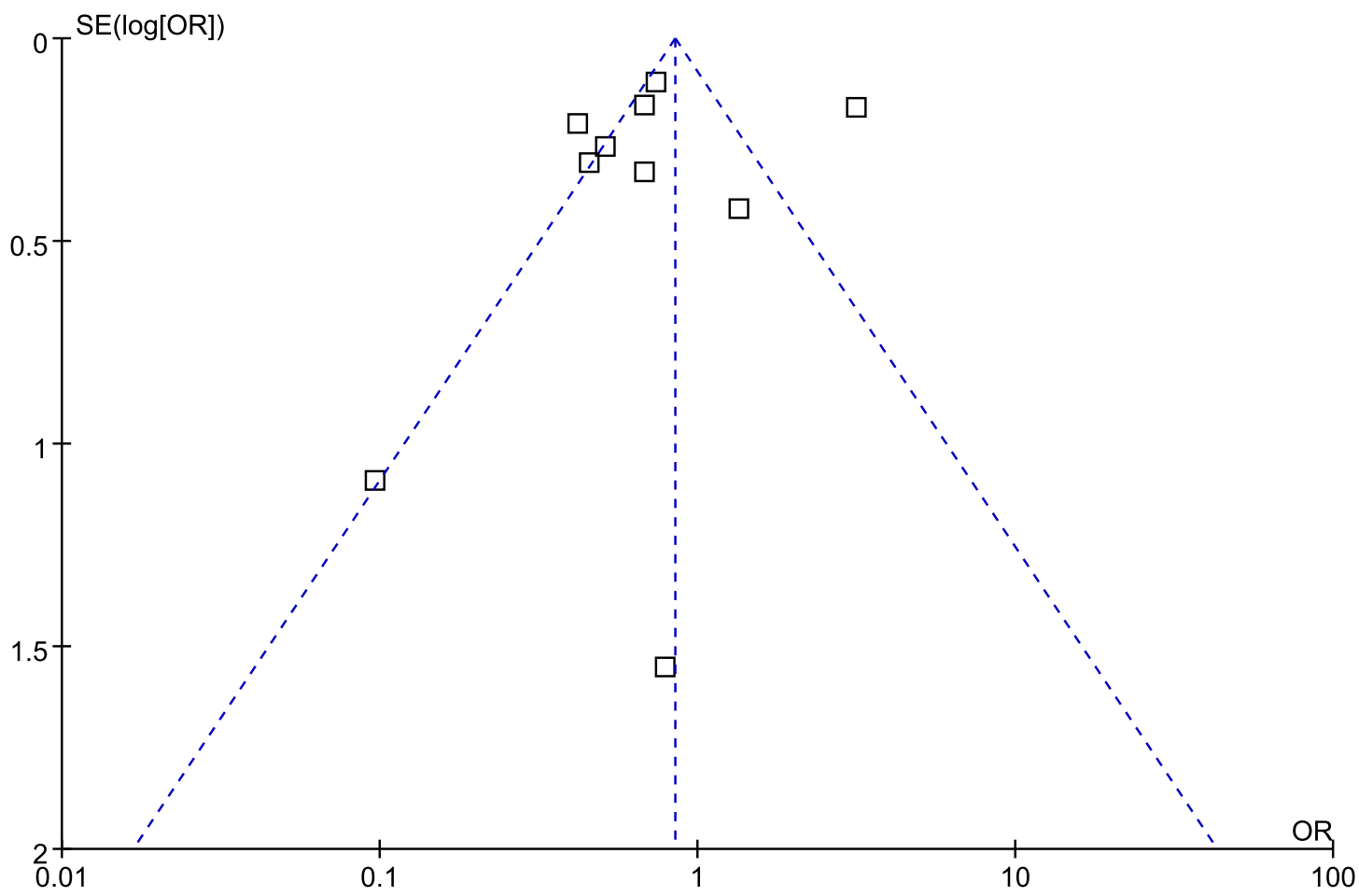

Supplement: Supplementary Materials — Supplementary Figure 6: funnel plot of meta-analysis of the association between KCNQ12237892 locus and T2DM under the allele model. Supplementary Figure 7: funnel plot of meta-analysis of the association between KCNQ12237892 locus and T2DM under the allele model (stratified analysis). Supplementary Figure 8: funnel plot of meta-analysis of the association between KCNQ12237892 locus and T2DM under the recessive model. Supplementary Figure 9: funnel plot of meta-analysis of the association between KCNQ12237892 locus and T2DM under the recessive model (stratified analysis). [file 6606830.f1.zip › Fig8 (1).pdf]

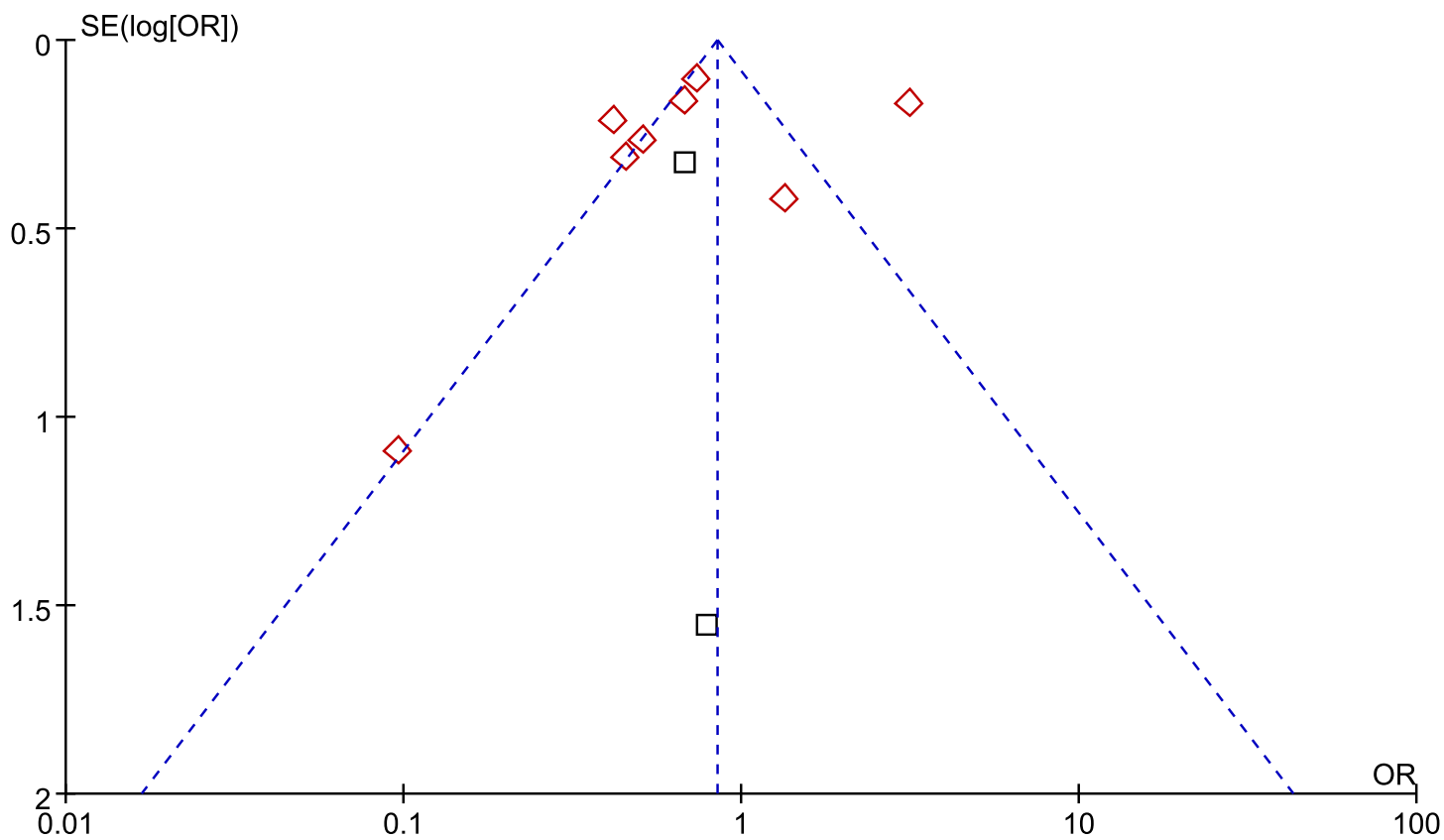

Supplement: Supplementary Materials — Supplementary Figure 6: funnel plot of meta-analysis of the association between KCNQ12237892 locus and T2DM under the allele model. Supplementary Figure 7: funnel plot of meta-analysis of the association between KCNQ12237892 locus and T2DM under the allele model (stratified analysis). Supplementary Figure 8: funnel plot of meta-analysis of the association between KCNQ12237892 locus and T2DM under the recessive model. Supplementary Figure 9: funnel plot of meta-analysis of the association between KCNQ12237892 locus and T2DM under the recessive model (stratified analysis). [file 6606830.f1.zip › Fig9 (1).pdf]
